# FXR antagonism of NSAIDs contributes to drug-induced liver injury identified by systems pharmacology approach

**DOI:** 10.1038/srep08114

**Published:** 2015-01-29

**Authors:** Weiqiang Lu, Feixiong Cheng, Jing Jiang, Chen Zhang, Xiaokang Deng, Zhongyu Xu, Shien Zou, Xu Shen, Yun Tang, Jin Huang

**Affiliations:** 1 Shanghai Key Laboratory of Regulatory Biology, The Institute of Biomedical Sciences and School of Life Sciences, East China Normal University, Shanghai 200241, China; 2 Shanghai Key Laboratory of New Drug Design, School of Pharmacy, East China University of Science and Technology, Shanghai 200237, China; 3 Department of Gynecology, Obstetrics and Gynecology Hospital of Fudan University, Shanghai 200011, China; 4 Shanghai Institute of Materia Medica, 555 Zuchongzhi Road, Shanghai 201203, China

## Abstract

Non-steroidal anti-inflammatory drugs (NSAIDs) are worldwide used drugs for analgesic, antipyretic, and anti-inflammatory therapeutics. However, NSAIDs often cause several serious liver injuries, such as drug-induced liver injury (DILI), and the molecular mechanisms of DILI have not been clearly elucidated. In this study, we developed a systems pharmacology approach to explore the mechanism-of-action of NSAIDs. We found that the Farnesoid X Receptor (FXR) antagonism of NSAIDs is a potential molecular mechanism of DILI through systematic network analysis and *in vitro* assays. Specially, the quantitative real-time PCR assay reveals that indomethacin and ibuprofen regulate FXR downstream target gene expression in HepG2 cells. Furthermore, the western blot shows that FXR antagonism by indomethacin induces the phosphorylation of STAT3 (signal transducer and activator of transcription 3), promotes the activation of caspase9, and finally causes DILI. In summary, our systems pharmacology approach provided novel insights into molecular mechanisms of DILI for NSAIDs, which may propel the ways toward the design of novel anti-inflammatory pharmacotherapeutics.

Non-steroidal anti-inflammatory drugs (NSAIDs) are commonly used agents for analgesic, antipyretic, and anti-inflammatory therapeutics^1^. However, NSAIDs often cause various adverse side effects (SE), such as drug-induced liver injury (DILI). Roughly 10% of total drugs that induced hepatotoxicity are related to NSAIDs^2^. Several NSAIDs, including ibufenac, bromfenac and benoxaprofen, have been withdrawn from the market due to hepatotoxicity^2^,^3^. In addition, nimesulide has never been approved or marketed in some countries because of highly frequent reports of severe liver damage^4^.

Several possible molecular mechanisms of liver damage induced by NSAIDs have been reported, including reactive metabolite, metabolic idiosyncrasy, impairment of ATP synthesis, and hyper-sensitivity^5^,^6^,^7^. However, the mechanism-of-action (MOA) of NSAIDs related to DILI has not been fully clarified. Systems pharmacology, an emerging research field of pharmacology, has open promising avenues to help researchers investigate the MOA of drugs^8^,^9^, understand molecular mechanisms of SE^10^,^11^, find new usages of old drugs (i.e. drug repositioning)^12^, and explore drug pharmacokinetics/pharmacodynamics (PK/PD) profiles^13^,^14^. The term of systems pharmacology currently describes a new research field of study by incorporating experimental and computational approaches to explore the complex drug MOA profiles, which would help scientists to explain both therapeutics and adverse SE of drugs.

In this study, we proposed a systems pharmacology approach to investigate the molecular mechanisms of NSAID-induced liver injury by systematically incorporating network analysis, molecular modeling, and *in vitro* assays (Figure 1). Specifically, network analysis and molecular modeling indicated that farnesoid X receptor (FXR) is a possible off-target protein mediating NSAID-induced liver injury. Furthermore, yeast two-hybrid and mammalian transactivation assays show that at least some NSAIDs (e.g. indomethacin) are potential FXR antagonists. The detailed molecular mechanisms of NSAID-induced liver injury are further explored experimentally. In summary, our systems pharmacology approach provided novel insights into the molecular mechanisms of NSAID-induced liver injury, which may be mediated through antagonism of FXR.

## Results

### Overview of the systems pharmacology approach

**Figure 1:**
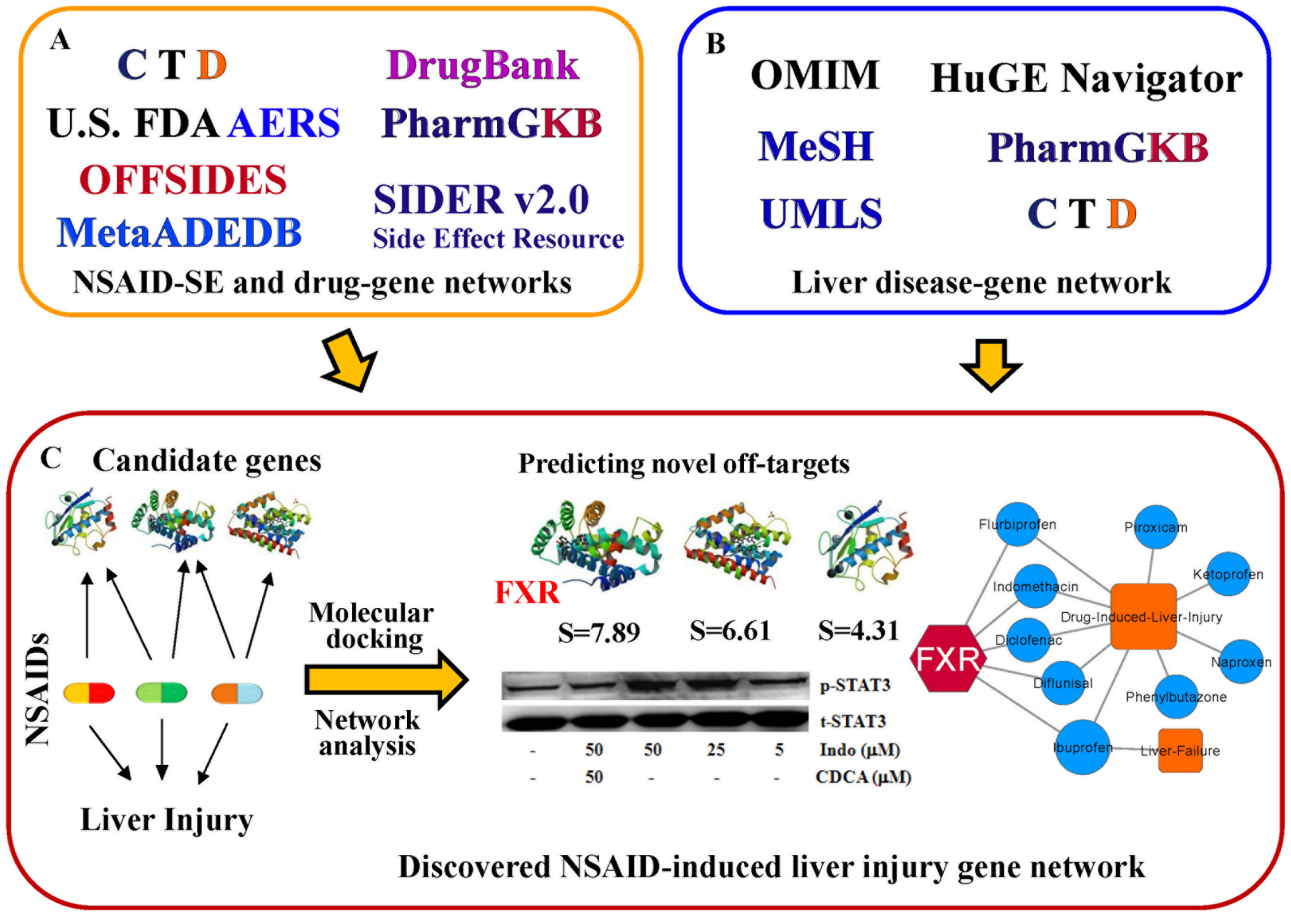
The diagram of a systems pharmacology approach. A systems pharmacology approach was developed to identify the mechanism-of-action of NSAIDs and investigate potential molecular mechanisms of NSAID-induced liver injury by incorporating network analysis, molecular modeling, and *in vitro* assays. (A) Construction of drug-SE association network by integrating data from five public databases. (B) Construction of liver gene-disease association network. (C) Inferring new candidate off-target proteins involved in NSAID-drug-induced liver injury, and validating its molecular mechanism using the *in vitro* assays. SE: side effects, NSAID: Non-steroidal anti-inflammatory drug.

**Figure 2:**
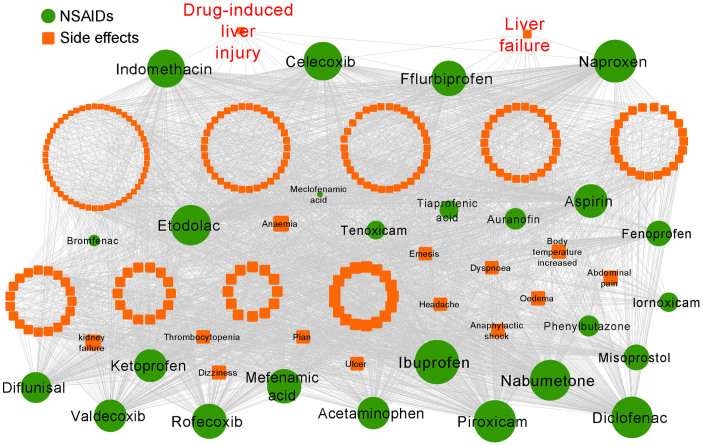
The NSAID-SE association network. In the network, nodes represent NSAIDs (Non-steroidal anti-inflammatory drug, green circles) and side effects (SE, gold squares) that were caused by at least 10 different NSAIDs, and where edges represent clinically reported drug-SE pairs. The size of NSAID and SE nodes is the number of NSAID-SE pairs (Degree). This graph and Figure 3 are prepared by Cytoscape (v2.8.3; http://www.cytoscape.org/).

We developed a systems pharmacology approach to identify the MOA of NSAIDs and investigate the potential molecular mechanisms of NSAID-induced liver injury by integrating the network analysis, molecular modeling, and *in vitro* assays (Figure 1). Specifically, we firstly collected a comprehensive NSAID-SE association dataset from five public databases: Comparative Toxicogenomics Database (CTD)^15^, SIDER^16^, OFFSIDES^17^,^18^, MetaADEDB^19^, and the U.S. Food and Drug Administration (FDA) Adverse Events Reporting System (AERS). In this study, only clinically reported drug-SE association pairs were used based on a previous study^19^. All NSAID and SE items were annotated using the most commonly used Medical Subject Headings (MeSH) or Unified Medical Language System (UMLS) vocabularies^20^. All duplicated drug-SE pairs were excluded. In total, 13,927 drug-SE pairs connecting 25 NSAIDs and 4,628 SE terms were obtained (Supplementary Table S1). We then built a high-quality NSAID-SE association network using a bipartite graph^12^, where nodes represent NSAIDs (green circles) and SE (gold squares) that was caused by at least 10 different NSAIDs, and where edges represent the clinically reported NSAID-SE associations (Supplementary Table S1). Figure 2 shows that DILI and liver failure are two high frequent adverse SE terms caused by multiple NSAIDs.

**Figure 3:**
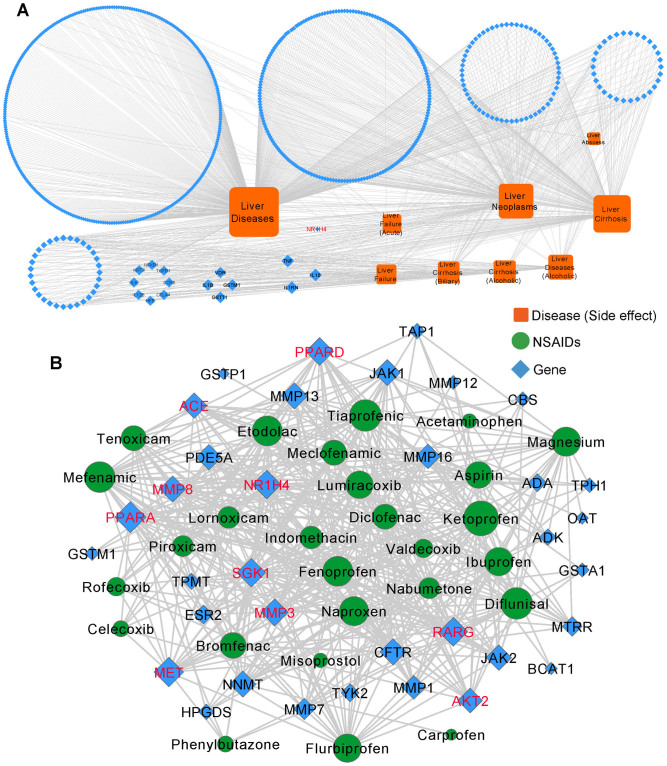
Identifying the NSAID-protein interaction network that is associated with drug-induced liver toxicity using the molecular modeling and network analysis. (A) Liver disease-gene association network, in which 9 liver disease terms (gold squares) and 627 genes (cyan diamonds) were connected if a gene is a known liver disease-associated genes annotated in four public databases. (B) Prediction of new NSAID-protein (off-target) interaction networks, in which a NSAID (green circle) and a protein encoded by the liver-disease associated gene are connected if the docking scores of NSAID-protein complex is higher than the average docking scores. 10 gene symbols labeled by red represent significantly predicted NSAID off-target proteins (*P* < 0.01). The detailed data are provided in Supplementary Tables S1 and S2.

Next, we constructed a gene-disease association network for 9 liver disease terms using the data from four public databases: the Online Mendelian Inheritance in Man (OMIM) database (December 2012)^21^, HuGE Navigator^22^, PharmGKB^18^, and CTD^15^ (Figure 1B). All liver disease terms were annotated using MeSH or UMLS vocabularies^23^, and the genes were further annotated using the Entrez ID and official gene Symbol based on the NCBI database (http://www.ncbi.nlm.nih.gov/). We excluded the computationally predicted and duplicated gene-disease pairs from different data resources. In total, 1,234 gene-disease pairs connecting 627 unique genes and 9 different liver disease terms were yielded for follow-up gene-disease association network building and network analysis (Figure 3A and Supplementary Table S1). Then, we incorporated the drug-SE and gene-disease networks, and used the molecular docking and network-based statistical analysis to predict candidate NSAID off-target proteins that are involved in liver diseases (i.e., DILI). Finally, we used the *in vitro* assays, including yeast two-hybrid assay, mammalian transactivation assay, quantitative real-time PCR (qRT-PCR), and western blot, to validate the predicted candidate off-target proteins experimentally and systematically investigate the molecular mechanisms of NSAID-induced liver injury.

### Inferring new candidate off-target proteins for NSAID-induced liver injury

We first used the molecular modeling approach to predict new candidate off-target proteins for NSAID-induced liver injury (Figure 1C). We searched the crystal structures for 627 liver disease-associated gene products (proteins) in the Protein Data Bank (PDB, http://www.rcsb.org/) database, and used the PISCES server^24^ to remove the redundant proteins and high similar proteins. We also removed 16 proteins which are known NSAID target proteins annotated in DrugBank^25^ and PharmGKB^18^. Additionally, proteins harboring PDB files that didn't have the known ligand-binding pockets with co-crystal small molecules were also excluded in order to improve the molecular docking accuracy. Finally, 28 NSAIDs were docked into 37 unique liver disease-associated proteins (Supplementary Table S2) using the Glide SP software (see Methods). To reduce false positive rate in the molecular docking process, we defined a high confidence NSAID-protein docked complex when docking score (glide gscore) of this complex is higher than average score (Supplementary Table S2). We defined a low confidence NSAID-protein docked complex when glide gscore of this complex is lower than the average score. As shown in Supplementary Table S2, we found 437 high and 476 low confidence NSAID-protein docked complexes, respectively. We then performed Fisher's exact test using the R package (v 3.0.1) to estimate the *P* values by ranking significant NSAID candidate off-target proteins using the glide gscores in Supplementary Table S2. The null hypothesis is that there is no significant association between the two category variables (high versus low confidence drug-protein docked complexes). The alternative hypothesis is that: if a protein has more docked complexes that are enriched in high confidence NSAID-protein docked complexes in comparison to the low confidence NSAID-protein docked complexes, this protein will more likely be a NSAID off-target.

We found that 10 proteins are significantly predicted to be NSAID off-targets (*P* < 0.01, Supplementary Table S2) using the above statistical model. We then built the drug-protein interaction network (Figure 3B) using a bipartite graph algorithm^12^, where nodes represent NSAIDs (green circles) and genes (cyan squares), and where edges represent that glide gscores of drug-protein complexes are higher than average scores (Supplementary Table S2). The red gene symbol shows that this gene product (protein) was significantly predicted to be a NSAID off-target (*P* < 0.01, Supplementary Table S2). Figure 3B shows 4 most significantly predicted NSAID off-targets: *PPARA* (*P* = 1.3 × 10^−7^), *RARG* (*P* = 1.3 × 10^−7^), *NR1H4* (*P* = 1.8 × 10^−4^), and *MET* (*P* = 1.8 × 10^−4^). FXR, encoded by gene *NRIH4*, is highly expressed in liver and other digestive organs, whose function is expended rapidly from initial roles in controlling metabolism of bile acids^26^, lipids^27^ and glucose^28^ to also regulating cell growth, fibrosis^29^, cirrhosis^30^, immunological responses^31^, inflammation and malignance^32^,^33^. There is increasing evidence to show that FXR plays a crucial role in liver regeneration and repair, and inflammatory responses, so hepatic FXR has caught more attention in treating liver related diseases (Figure 3A). Based on the molecular docking, network-based statistical analysis, and literature data, we hypothesized that a predicted off-target protein of NSAIDs, FXR (*P* = 1.8 × 10^−4^), may contribute to DILI.

### *In vitro* assays of FXR-antagonizing NSAIDs using yeast two-hybrid assays

Yeast two-hybrid system is a powerful tool for identifying potential agonist or antagonist of nuclear receptor based on the protein-protein interaction between nuclear receptor and its transcriptional co-activator^34^. Based on the predicted results through our systems pharmacology approach, the yeast two-hybrid system was set up according to a previous study^35^ to test whether NSAIDs can modulate the activation of FXR. A series of NSAIDs with diverse chemical structures were collected and experimentally evaluated for their effects on the interaction between FXR and its co-activator SRC1. Compounds with inhibition rates larger than 50% at 10 μM were further quantitatively measured by the IC_50_ values. Table 1 reveals that at least some NSAIDs exhibit potent antagonistic activities on FXR. Seven tested compounds display inhibition rates above 60% at 10 μM, with IC_50_ values ranging from 0.7 to 8.48 μM. Phenylbutazone and flurbiprofen indicated the highest FXR antagonist activities with IC_50_ values of 0.7 and 0.78 μM, respectively. In addition, ibuprofen, fenoprofen, flurbiprofen, indomethacin, diclofenac and phenybutazone were more potent FXR antagonist than guggulsterones (GS), a well-known natural occurring steroidal FXR antagonist (IC_50_ = 6.47 μM)^36^. However, oxicams and coxibs exhibited weak antagonistic activity on FXR. The previous studies reported an immune-mediated mechanism that is related to liver toxicity induced by oxicams and coxibs^37^,^38^. Thus, the weak antagonistic activities of oxicams and coxibs on FXR are consistent with the previous studies^37^,^38^. Interestingly, none of the NSAIDs exhibited significant agonistic activities on FXR.

### Effects of NSAIDs on transcriptional activity in FXR transactivation assay

**Figure 4:**
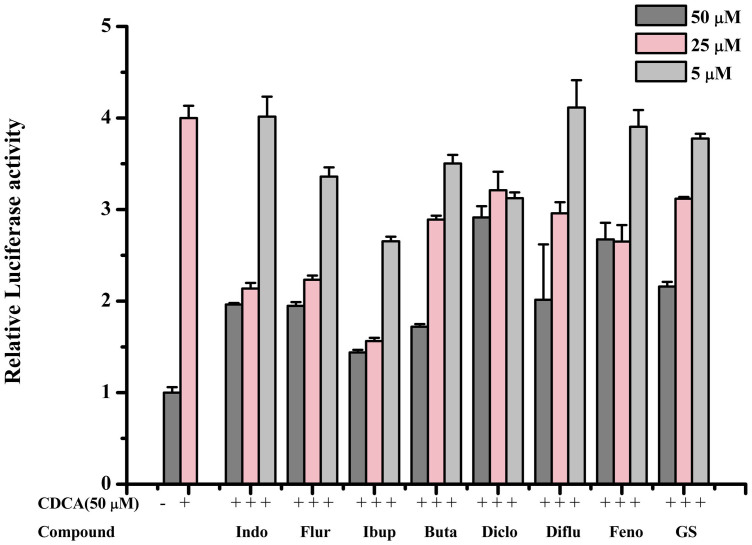
Concentration-response effects of NSAIDs on FXR transactivation inhibition induced by CDCA. The luciferase reporter assay was carried out in 293T transiently transfected with pCMX-FXRα, pCDNA3.1-RXRα, luciferase reporter plasmid pGL3-FXRE-Luc and a Renilla luciferase expression plasmid (as transfection control) in mammalian transactivation assays. The system was stimulated with 50 μM CDCA alone or in combination with different concentrations of NSAIDs (indomethacin was indicated as Indo, flurbiprofen was indicated as Flur, ibuprofen was indicated as Ibup, phenylbutazone was indicated as Buta, diclofenac was indicated as Diclo, diflunisal was indicated as Diflu, fenoprofen was indicated as Feno). Data are means of three experiments carried out in triplicate.

In order to further investigate the effects of NSAIDs on FXR transcription activity, we performed the mammalian transactivation assays. HEK293T cells were co-transfected with a FXR response element carrying a luciferase reporter plasmid and expression vectors for FXR and retinoid X receptor (RXR) together with a pRL-SV40 control vector. Cells were treated with different drugs at the concentrations indicated. The transient transfection assays show that seven drugs with inhibition rates larger than 60% in yeast two-hybrid display excellent antagonistic activity. As shown in Figure 4, ibuprofen is more potent than GS and decreases transcriptional activity on FXR induced by chenodeoxycholic acid (CDCA) via a dose-dependent manner. The transcription inhibition activity of indomethacin, diflunisal, flurbiprofen and phenybutazone are similar to positive control GS. However, diclofenac and fenoprofen display less antagonistic activity than GS.

### Indomethacin and ibuprofen induce FXR target genes in HepG2 cells

**Figure 5:**
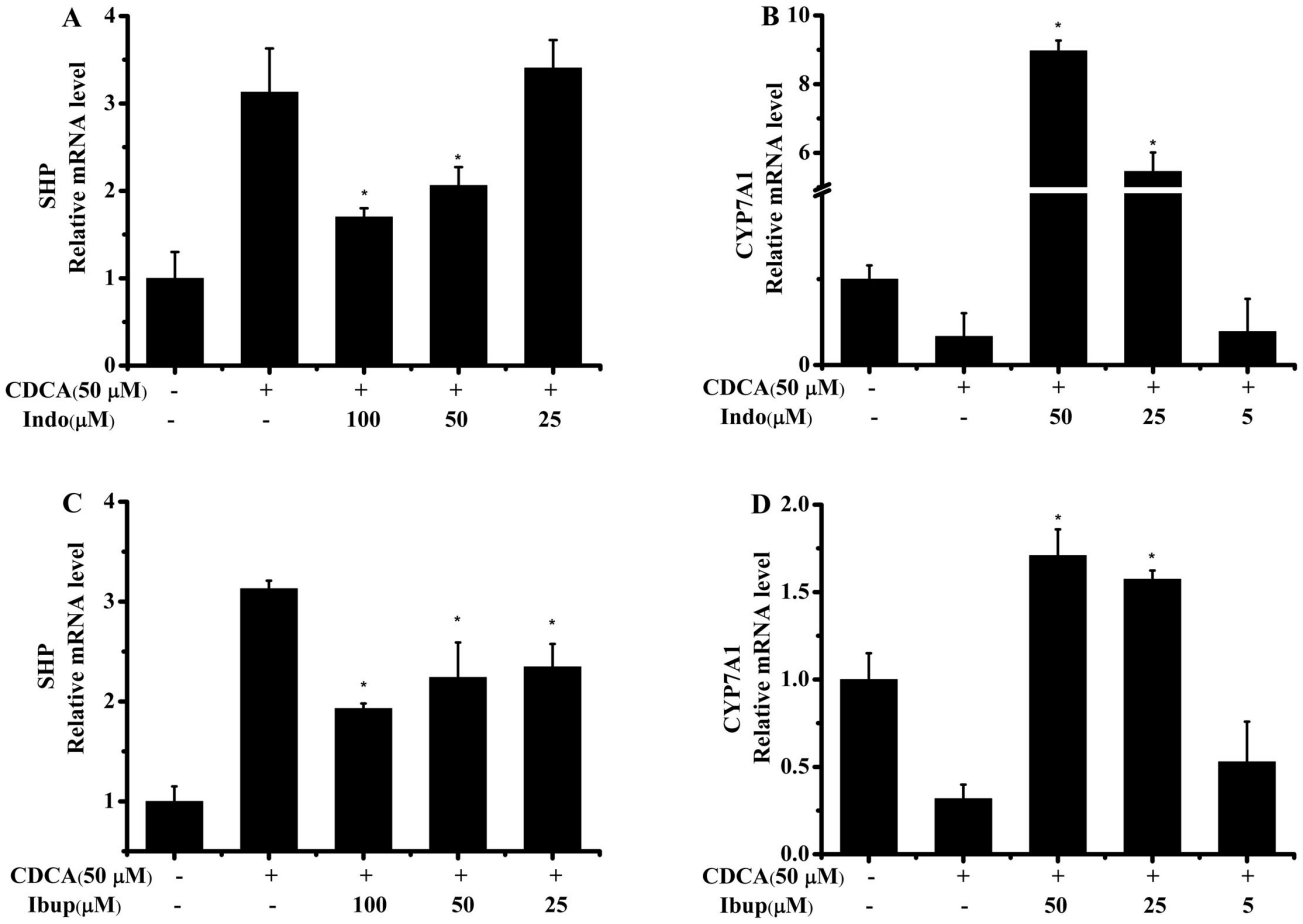
Indomethacin and ibuprofen regulate FXR downstream target gene mRNA expression in HepG2 cells. HepG2 cells were incubated with 50 μM CDCA alone or in combination with different concentrations indomethacin (A,B) or ibuprofen (C,D) for 24 h and cDNA synthesized from isolated mRNA served as template in qPCR experiments. The data were carried out in at least three independent experiments. Significance was determined by t test (*P < 0.05 versus cells treated with CDCA).

To further examine FXR antagonistic activity of NSAIDs, two most effective antagonists (indomethacin and ibuprofen) were selected to test their effects on the expression of genes targeted by CDCA in an FXR-dependent manner. We incubated HepG2 cells with different concentrations of indomethacin and ibuprofen for 24 h. The mRNA levels of the FXR target genes were quantified by qRT-PCR. Figure 5 shows that indomethacin and ibuprofen inhibit the expression of canonical FXR target gene SHP in a dose dependent manner. Furthermore, indomethacin and ibuprofen obviously increase the expression of CYP7A1 that is negatively regulated by FXR in a SHP-dependent manner. Additionally, indomethacin displays a higher biological activity than ibuprofen on genes *SHP* and *CYP7A1* both of which are regulated by FXR. Put together, indomethacin and ibuprofen, as potent FXR antagonists, could inhibit transcriptional activity on FXR/RXR heterodimer and negatively regulate FXR target genes *SHP* and *CYP7A1*.

### Indomethacin could induce STAT3 phosphorylation in HepG2 cells

**Figure 6:**
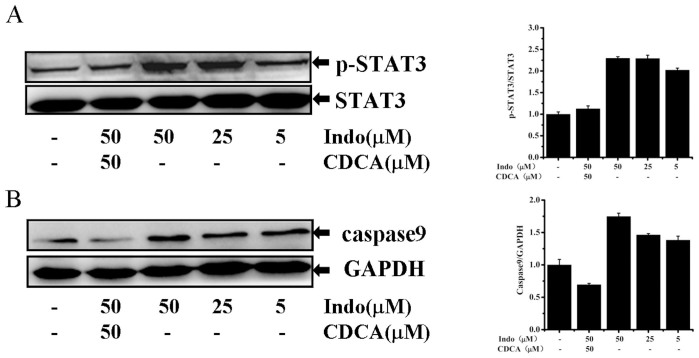
Indomethacin induce liver injury related biochemical events in HepG2 cell. (A) Indomethacin induces STAT3 phosphorylation in a dose-dependent manner. The HepG2 cells were grown to about 70% to 80% confluence in complete medium and then cultured in low serum medium in the presence of CDCA or indomethacin at the concentration indicated. The phosphorylated STAT3 was determined by western blot analysis. (B) CDCA could suppress indomethacin-induced caspase9 activation. HepG2 cells were treated with indomethacin or CDCA for indicated concentrations and the protein level of caspase9 and GAPDH were detected by western blot.

Lipopolysaccharides (LPS) stimulated animal model indicated that liver injury could lead to the signal transducer and activator of transcription 3 (STAT3) phosphorylation and inhibit excessive STAT3 activation^32^. It has also been reported that liver injury caused by indomethacin in PiZ mice is due to increasing activation of caspase9 and increasing hepatocellular proliferation^39^. In subsequent experiments, we further investigated whether NSAIDs could induce STAT3 activation and suppress the expression of caspase9. We selected indomethacin to conduct the follow-up experiments due to its high antagonistic activity on FXR (Figure 5). We found that indomethacin significantly increases the STAT3 phosphorylation level in HepG2 cells, whereas CDCA pretreatment suppresses indomethacin-induced STAT3 activation (Figure 6A). The previous study showed that CDCA could attenuate hepatocyte inflammatory damage and was accompanied by inhibition of STAT3 phosphorylation^32^. Thus, indomethacin may disrupt the protective effects of FXR through the activation of STAT3 phosphorylation. From Figure 6B, we found that indomethacin significantly stimulates caspase9 expression in a dose-dependent manner. However, the caspase9 levels were substantially attenuated back to the normal level if co-treated with indomethacin and CDCA. In summary, these results indicate that indomethacin induces similar liver injury-related biochemical reactions in HepG2 cell.

## Discussion

NSAIDs are among the most commonly prescribed agents in clinical practices for analgesic, antipyretic, anti-inflammatory, and rheumatological disorder therapeutics. However, NSAIDs often cause serious liver injury^40^,^41^, which leads to increasing health care costs. Moreover, the molecular mechanism of NSAID-induced liver injury is not clear so far. To address this important issue, we developed an integrative systems pharmacology approach to investigate the molecular mechanisms of NSAID-induced liver injury. The molecular modeling and network-based statistical analysis revealed that FXR is a candidate off-target protein for NSAIDs. The yeast two-hybrid assay then confirmed that NSAIDs display strong inhibitive activities on FXR. Furthermore, the mammalian transactivation assay and qRT-PCR assay implied that NSAIDs could decrease FXR transcriptional activity induced by CDCA as a dose-dependent manner, and negatively regulate FXR target genes. Finally, the western blot assays showed that indomethacin activate STAT3 by increasing STAT3 phosphorylation in HepG2 cells. Interestingly, the STAT3 levels could attenuate back to the normal level if the cells were treated with CDCA and indomethacin simultaneously. In summary, our systems pharmacology approach provides novel insights into the molecular mechanisms of NSAID-induced liver injury, which is mediated through antagonism of FXR.

In the past decade, several potential molecular mechanisms of liver damage induced by NSAIDs were reported, including reactive metabolite, metabolic idiosyncrasy, impairment of ATP synthesis, and hyper-sensitivity^5^,^6^,^7^. In this study, we found a novel molecular mechanism that FXR antagonism of NSAIDs causes DILI by uniquely integrating computational approach and *in vitro* assays. Although the IC_50_ value of NSAIDs for antagonism of FXR is at the micromolar level, these concentrations are within the therapeutic range when using recommended therapeutic dosing in humans^42^,^43^. FXR, a ligand-activated transcription factor, which highly expressed in liver and other digestive organs, plays an important role to protect cells against bile acid induced toxicity^44^. In addition, FXR not only is a master regulator of bile acid homeostasis, but also mediates liver inflammatory and liver regeneration/repair^45^,^46^. Activation of FXR affects hepatitis B virus DNA replication as well as prevents hepatocarcinogenesis by regulating the NF-κB signaling pathway to inhibit the injury caused by the persistent immune response and cytotoxicity induced by the virus products and accumulation of toxic bile acids^47^. Hayakawa et al. found a potential mechanism that FXR regulates the proliferation of hepatocellular carcinoma cells^48^. He et al. demonstrated a potential therapeutic role of FXR agonists in relieving LPS-induced liver inflammatory injury^32^. Recently, Kumagai et al. pointed out the clinicopathological significances of FXR expression in hepatic cell carcinoma (HCC) patients, and further indicated that enhanced expression of FXR in HCC had a close association with both proliferative activity and therapeutic modality^49^. Collectively, these findings show that FXR plays a crucial role during the progression of liver related disease. In addition, FXR agonist has been shown to protect against LPS-mediated liver inflammatory injury, CCl_4_-induced toxic injury, cholestatic liver injury and fibrosis^50^,^51^. Thus, FXR antagonism of NSAIDs contributing to DILI is consistent with the previous studies^7^,^52^,^53^,^54^,^55^.

FXR activation down-regulated *CYP7A1* and reduced hepatic bile acid levels to protect the liver from apoptosis and necrosis^56^. Without FXR, liver is prone to enter endless cycles of injury that produces inflammatory cytokines. In this study, we further selected two high potent FXR antagonists: indomethacin and ibuprofen, to examine whether NSAIDs could induce FXR target gene expression using qRT-PCR assays. Our results indicated that both indomethacin and ibuprofen up-regulate *CYP7A1* expression (Figure 5), which may cause hepatic bile acid level increasing and further induce liver injury. Xu et al. demonstrated that the activation of *STAT3* is an important effect in LPS-induced liver inflammation^32^. Moreover, liver injury was combined with increasing hepatocellular proliferation and activation of caspase9^39^. Here, we further found that indomethacin could stimulate *STAT3* and caspase9 activation in a dose-dependent manner. CDCA, the natural occurring agonist of FXR, was able to obviously decrease the phosphorylation of *STAT3* and caspase9 levels induced by indomethacin (Figure 6). Although the activation of *STAT3* and caspase9 were observed in vitro, indomethacin may also cause similar biochemical event in the progress of liver injury in vivo. Collectively, we proposed that NSAID-induced liver injury is at least partially mediated through antagonism of FXR.

Systems pharmacology under systems biology framework has been successfully used to investigate the MOA of drugs^8^,^9^,^57^,^58^, understand molecular mechanisms of SE^10^, find new usages of existing drugs (i.e. drug repositioning)^12^,^59^. Yang et al. utilized the molecular docking and chemical-protein interactome analysis to study the molecular mechanism of life-threatening agranulocytosis caused by clozapine^11^. They found that *HSPA1A* is a potential off-target of clozapine, which is associated with agranulocytosis. Cheng et al. studied the polypharmacological profiles of drugs by integrating drugs' chemical, side effect, and therapeutic space under systems pharmacology framework^9^. They found the new MOA of three approved antipsychotic drugs, which is involved in extrapyramidal side effects, tardive dyskinesia, endocrine disorder, galactorrhea, and amenorrhea. In this study, we further developed an integrative systems pharmacology approach by uniquely incorporating molecular docking and network-based statistical analysis (including drug-SE association, gene-disease association, and drug-gene interaction networks). Moreover, we experimentally validated that FXR antagonism of NSAIDs is a potential molecular mechanism of DILI, using yeast two-hybrid assay, mammalian transactivation assays, qRT-PCR, and western blot analyses. However, there are several potential limitations in the current systems pharmacology approach. First, both of gene-disease association and drug-SE association network from public databases are far from completeness. Second, we used the molecular docking to predict the potential off-target proteins for NSAIDs. However, the current protein three-dimensional structures are far from completeness and the molecular docking approaches may not be accurate due to the feasibility of protein structures^60^. In addition, the liver disease-associated protein X-ray crystal structures used in current study are a limited representation of the entire human liver disease-associated proteome. In the future, we may improve our systems pharmacology approach in the following ways: (i) integrate the toxicogenomics data, such as ToxCast^61^ and DrugMatrix (https://ntp.niehs.nih.gov/drugmatrix/index.html), to build a high-quality, comprehensive drug-gene-disease network; (ii) develop novel statistical-based or network-based algorithms^57^,^62^ to replace the regular molecular docking method to identify the MOA of drugs. Despite its limit on the current systems pharmacology approach, this study represents a useful computational and experimental approach under systems pharmacology perspective to identify the unknown molecular mechanism of NSAID-induced liver injury. We successfully identified a novel molecular mechanism underlying the liver injury caused by NSAIDs, which is mediated through antagonism of FXR. In conclusion, our systems pharmacology approach provided a useful strategy to explore the complex MOA of drugs and would have potential implications toward understanding the unknown molecular mechanisms of DILI, which may propel the new ways toward the design of novel anti-inflammatory therapeutics by reducing the liver injury.

## Methods

### Construction of drug-SE association network

The original data were downloaded from five public drug-SE associated databases: CTD^15^, SIDER^16^ (version 2) and OFFSIDES^17^,^18^, MetaADEDB^19^, and U.S. FDA Adverse Events Reporting System (AERS) from http://www.fda.gov/Drugs/GuidanceComplianceRegulatoryInformation/Surveillance/AdverseDrugEffects/ucm083765.htm. All NSAID and SE items were annotated with the most commonly used MeSH or UMLS vocabularies (2012 release, xml format) downloaded from the website of National Library of Medicine (http://www.nlm.nih.gov/mesh/gcm.html). Only data points with clinically reported evidence were used, and the duplicated pairs were excluded. In order to improve the quality of drug-SE association network, we only visualized the high frequent SE terms that are caused by at least 10 different NSAIDs in Figure 2. In addition, we also collected known drug-target interactions from DrugBank^25^ and PharmGKB^18^.

### Construction of liver disease-gene association network

We collected liver disease-gene association data from four public databases: OMIM database^21^ (December 2012), HuGE Navigator^22^, PharmGKB^18^, and CTD^15^. Here, all genes were annotated using gene Entrez ID and official gene symbols based on the NCBI database (http://www.ncbi.nlm.nih.gov/). Herein, 9 liver disease terms were used and annotated using MeSH or UMLS vocabularies^23^ (Supplementary Table S1). The computationally predicted gene-disease pairs and duplicated pairs were removed. In total, 1,234 gene-disease pairs connecting 627 unique genes and 9 different liver disease terms were obtained for network analysis (Supplementary Table S1).

### Inferring new candidate off-target proteins for NSAID-induced liver injury

Here, we used the molecular docking method to predict putative off-target proteins that are involved in NSAID-induced liver injury. We mapped the proteins encoded by above 627 liver disease-associated genes into the PDB database (http://www.rcsb.org/), and used the PISCES server^24^ to remove the redundant proteins and high similar sequence identify proteins. In this study, the percentage sequence identity cutoff is 20%, the resolution cutoff is 1.8 angstroms, and the R-factor cutoff is 0.25. Additionally, the PDB files with the unknown ligand pockets were also excluded to improve the molecular docking accuracy. Based on these criteria, 37 unique liver disease-associated proteins with known ligand-protein PDB complexes were yielded for molecular docking experiments.

Two-dimensional chemical structures of 28 NSAIDs were downloaded from the DrugBank^25^ and were prepared using LigPred 2.5 implemented in Maestro, version 9.3 (http://www.schrodinger.com/). After that these crystal structures with ligands were submitted to Schrodinger's Protein Preparation Wizard workflow in Maestro, version 9.3 and prepared carefully. Specifically, bond orders and charges were thus assigned, and the orientation of hydroxyl groups, amide groups of Asparagine and Glutamine, and the charge state of Histidine residues were optimized. Energy minimization was carried out using MacroModel 9.9 with default setting. During this process, the OPLS_2005 force field was chosen and the possible ionization states of each NSAID at the pH range of 5.0–9.0 were generated. All docking calculations were run in the “Standard Precision” (SP) mode in Glide 5.8, and the center of the grid box was set to the original PDB ligand coordinates with the size of 10 Å. At most 5 poses were written out for each ligand. All other parameters were left at default settings. The Glide scoring function (Glide gscore) was used to select the final top 5 poses for each ligand and the poses with the highest Glide gscore value were kept for further network analysis.

### Reagents

The restriction enzymes were obtained from New England Biolabs (Beijing, China). *p*-nitrophenyl α-D-galactopyranoside, guggulsterone, CDCA, yeast nitrogen base without amino acids, dimethyl sulfoxide (DMSO) and glucose were all purchased from Sigma (Shanghai, China). The dropout supplement free from leucine and tryptophan (-Leu/-Trp DO supplement) and quantitative real-time PCR kit were bought from Takara (Dalian, China). Dulbecco's modified Eagle's Medium (DMEM) and fetal bovine serum (FBS) was from Gbico (Shanghai, China). 293Fect was purchased from Pregene (Beijing, China). Dual-Luciferase Reporter Assay System was obtained from Promega (Beijing, China). RNA extraction reagent and reverse transcription kit were purchased from Toyobo (Shanghai, China). Rabbit anti-phosphorylated-STAT3, rabbit anti-total-STAT3 and caspase9 were from Bioworld Technology, Inc (Nanjing, China). GAPDH antibody was obtained from Kangcheng Bio-tech (Shanghai, China). All NSAIDs were purchased from Sigma.

### Plasmids

The yeast expression plasmids pGADT7 and pGBKT7 were from Clontech (Palo Alto, CA). Human FXRα-LBD (amino acids 200–473) was subcloned into vector pGBKT-7 using NdeI and BamHI restrict enzyme sites. The primers used for PCR amplification were listed as follows: FXRα-LBD (sense) 5′-ATCATATGGAAATTCAGTGTAAATCTAAGCG-3′, (anti-sense) 5′-ATGGATCCTCACTGCACGTCCCA-3′. The combination plasmid pGADT7-SRC1 was prepared as described previously^63^. The luciferase reporter plasmid pGL3-FXRE-Luc was generously donated by Dr. Majlis Hermansson (AstraZeneca R&D Mölndal, Sweden).

### Yeast two-hybrid assay

A yeast two-hybrid system for FXRα was constructed by yeast co-transformation with pGBKT7-FXR LBD and pGADT7-SRC1 according to manufacturer's protocols. After co-transforming the two constructs into yeast strain AH109, we successfully evaluated FXRα/SRC1 interactions by conducting a convenient α-galactosidase assay. Yeast transformants were grown in -Leu, -Trp selection media enriched with DMSO or indicated compounds in hFXR agonist testing, and in antagonist assays treated with tested compounds plus 10 μM CDCA. 24 h later, we harvested the yeast cells and analyzed α-galactosidase activity using *p*-nitrophenyl α-D-galactopyranoside as the substrate. The α-galactosidase activity was calculated according to the following formula: α-galactosidase activity [milliunits/(mL × cell)] 
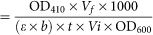
where *t* is the elapsed time of incubation (min), *V_f_* is the final volume of assay (200 μL), *V_i_* is the volume of culture medium supernatant added (16 μL), OD_600_ is the optical density of overnight culture, and *ε* × *b* is the *p*-nitrophenol molar absorptivity at 410 nm × the light path (cm) = 10.5 mL/μmol.

### Mammalian transactivation assays

293T cells were maintained in DMEM supplemented with 10% fetal bovine serum at 37°C in 5% CO_2_. Cells were seeded in a 24-well plate and transfected with the plasmid pCMX-FXRα, pCDNA3.1-RXRα, and luciferase reporter plasmid pGL3-FXRE-Luc. pRL-SV40, containing a Renilla luciferse gene for normalizing transfection was also co-transfected. Transfection was mediated by 293Fect according to manufacturer's instructions. After 12 h, the transfection medium was removed and cells were incubated with culture medium containing indicated compounds or indicated compounds with 50 μM CDCA. 24 h later, the luciferase activity was measured using Dual-Luciferase Reporter Assay System.

### Quantitative real-time PCR (qRT-PCR)

HepG2 cells were cultured at 37°C in DMEM media supplemented with 10% FBS. And then cells were seeded into 6-well plates and grew to 80–90% confluence. After incubation with the test compounds for 24 h, cells were harvested and total RNA was isolated using Trizol reagent. The first single-strand cDNA was synthesized using reverse transcriptase kit. The qRT-PCR was performed using Bio-Rad CFX96^TM^ real time PCR system according to the manufacturer's instruction with specific primers for the FXR target gene SHP (forward 5′-CCCAAGATGCTGTGACCTTT-3′, reverse 5′-CCAGAAGGACTCCAGACAGC-3′), CYP7A1 (forward 5′- GAGAAGGCAAACGGGTGAAC-3′, reverse 5′- GCACAACACCTTATGGTATGACA-3′) for detection of the transcripts. Transcription levels were normalized to GAPDH mRNA levels (forward 5′- GAAGGTGAAGGTCGGAGT-3′, reverse 5′- CATGGGTGGAATCATATTGGAA-3′). The relative RNA quantities were calculated using the comparative threshold cycles (Ct) method. The Ct for the SHP, CYP7A1 and GAPDH signals were determined in triplicate experiments.

### Western blot

For STAT3 phosphorylation and caspase9 expression analysis, HepG2 cells were seeded in 6-well plates and then treated with varied compounds in serum-free DMEM media. After 24 h incubation, cells were washed twice with ice-cold phosphate buffered saline (PBS) and lysed in RIPA buffer including 5 mM NaF, 1 mM PMSF, 2 mM NaVO_4_ and protease inhibitor cocktail. Lysates were then centrifuged at 10,000 rpm for 10 min at 4°and the protein concentrations were determined by BCA assay. Whole cell extracts was resolved on 10% SDS-PAGE and transferred to polyvinylidene fluoride (PVDF) membrane. The membrane was blotted with antibodies against p-STAT3, total-STAT3, caspase9 and GAPDH, and then examined by ECL detection system according to the manufacturer's instructions (Amersham Pharmacia Biotech).

### Network analysis and statistical analysis

We analyzed and built the network graph using the Cytoscape (v2.83, http://www.cytoscape.org/). All experiments were performed at least three independent determinations for each group. The differences between groups were evaluated for statistics significance by Student's t-test (*P* < 0.05 was considered to be statistically significant). All statistical tasks were performed in R package (v3.0.1; http://www.r-project.org/).

## Author Contributions

J.H. and Y.T. conceived and directed the projects. W.L., F.C. and J.J. designed the study, and performed the experiments. S.Z., X.D., Z.X., C.Z. and X.S. participated in data analysis and model building. F.C., W.L., J.J., J.H. and Y.T. wrote the manuscript.

## Supplementary Material

Supplementary Information Supplementary Table S1

Supplementary Information Supplementary Table S2

## Figures and Tables

**Table 1:** The antagonistic activities of NSAIDs against Farnesoid X Receptor (FXR) using yeast two hybrid assays

Classification	Drug name	FXR activation (10 μM)	FXR inhibition (10 μM)	IC_50_ (μM)
Salicylates	Aspirin	1.12	41.68%	NS
	Diflunisal	1.31	93.32%	8.48
Propionic acid derivatives	Ibuprofen	1.27	96.20%	1.08
	Fenoprofen	1.27	63.35%	5.59
	Flurbiprofen	1.65	94.98%	0.78
Acetic acid derivatives	Indomethacin	0.99	66.27%	5.76
	Diclofenac	1.24	88.66%	1.71
Enolic acid derivatives(Oxicams)	Piroxicam	1.13	30.69%	NS
	Tenoxicam	1.05	16.90%	NS
Selective COX-2 inhibitors (Coxibs)	Celecoxib	1.12	28.30%	NS
	Rofecoxib	1.18	39.90%	NS
Pyrazolones	Phenylbutazone	0.99	91.88%	0.7
Control	GS	ND	60.72%	6.47
	CDCA	2.7	ND	ND
	DMSO	1	0	ND

Data represents average values of at least triplicate measurements determined by yeast two hybrid assays. This system employs the interaction between hFXR-LBD and the coactivator SRC1. The inhibition rate (%) was calculated using the equation: [(GA_CDCA_-GA_treated_)/(GA_CDCA_-GA_DMSO_)] × 100%, GA indicates α-galactosidase activity. Attempts to determine IC_50_ values were made if the inhibition rate at 10 μM was larger than 50%. ND: not determined. NS: not significance, IC_50_ > 25 μM.
